# Comparison of a Prototype SARS-CoV-2 Lateral Flow IMMUNOASSAY with the BinaxNOW^TM^ COVID-19 Antigen CARD

**DOI:** 10.3390/v14122609

**Published:** 2022-11-23

**Authors:** Haydon J. Hill, Timsy Uppal, Derrick Hau, Sujata G. Pandit, Jose Arias-Umana, Abigail J. Foster, Andrew Gorzalski, Kathryn J. Pflughoeft, Amanda R. Burnham-Marusich, Dana E. Reed, Marcellene A. Gates-Hollingsworth, Lynette Gumbleton, Subhash C. Verma, David P. AuCoin

**Affiliations:** 1 Department of Microbiology and Immunology, Reno School of Medicine, University of Nevada, Reno, NV 89557, USA; 2 Nevada State Public Health Laboratory, Reno, NV 89503, USA; 3 DxDiscovery Incorporated, Reno, NV 89557, USA

**Keywords:** SARS-CoV-2, COVID-19, antigen assays, lateral flow immunoassay (LFI), monoclonal antibodies (mAb)

## Abstract

Severe acute respiratory syndrome coronavirus 2 (SARS-CoV-2) is the virus responsible for the COVID-19 pandemic. From the onset of the pandemic, rapid antigen tests have quickly proved themselves to be an accurate and accessible diagnostic platform. The initial (and still most commonly used antigen tests) for COVID-19 diagnosis were constructed using monoclonal antibodies (mAbs) specific to severe acute respiratory syndrome coronavirus (SARS-CoV) nucleocapsid protein (NP). These mAbs are able to bind SARS-CoV-2 NP due to high homology between the two viruses. However, since first being identified in 2019, SARS-CoV-2 has continuously mutated, and a multitude of variants have appeared. These mutations have an elevated risk of leading to possible diagnostic escape when using tests produced with SARS-CoV-derived mAbs. Here, we established a library of 18 mAbs specific to SARS-CoV-2 NP and used two of these mAbs (1CV7 and 1CV14) to generate a prototype antigen-detection lateral flow immunoassay (LFI). A side-by-side analysis of the 1CV7/1CV14 LFI and the commercially available BinaxNOW^TM^ COVID-19 Antigen CARD was performed. Results indicated the 1CV7/1CV14 LFI outperformed the BinaxNOW^TM^ test in the detection of BA.2, BA.2.12.1, and BA.5 Omicron sub-variants when testing remnant RT-PCR positive patient nasopharyngeal swabs diluted in viral transport media.

## 1. Introduction

Severe acute respiratory syndrome coronavirus-2 (SARS-CoV-2) is the causative agent of COVID-19 [1]. As of 2 September 2022, the virus has caused an estimated 600 million infections and over 6.4 million deaths [2]. To contain the pandemic, the FDA has approved vaccines, therapeutics, and diagnostics in record time [3,4,5]. While this has served to slow hospitalizations and deaths, viral transmission has not been halted. Data has shown that social distancing and quarantining of infected individuals is one of the most effective ways to combat the spread of SARS-CoV-2 [6]. In order to effectively quarantine, broad availability of accurate and rapid diagnostic tests is critical.

SARS-CoV-2 is a positive-sense single-stranded RNA virus that encodes for multiple genes that are expressed following infection. Various viral targets are used for diagnosing SARS-CoV-2 infections. Nucleic acid amplification tests (NAATs), such as polymerase chain reaction (PCR), primarily detect viral genomic RNA that encodes the spike and nucleocapsid proteins [7]. Antigen tests, such as lateral flow immunoassays (LFIs), primarily detect viral proteins such as the nucleocapsid protein (NP).

The gold standard diagnostic for SARS-CoV-2 infection are NAATs [8]. However, RNA viruses have a rapid mutation rate, and SARS-CoV-2 is no exception. B.1.617.2 (Delta) and B.1.1.529 (BA.1, Omicron) have developed many mutations, which can result in immune evasion [9,10,11]. The cumulative mutations acquired by the Delta and Omicron variants resulted in 9- and 36- amino acid replacements, respectively, in the spike protein alone, in reference to the original WA1 strain [12,13]. Because NAATs use nucleic acid primers to recognize and amplify their targets, inclusion of one or more base changes in the targeted nucleotide sequence is potentially enough to inhibit the reaction. The proliferative mutations acquired by the Omicron variant were enough to reduce clinical sensitivity with certain NAATs, leading to diagnostic escape. As a result, the Food and Drug Administration (FDA) ultimately recommended against using two NAATs for SARS-CoV-2 detection [14,15,16].

In general, lateral flow immunoassays (LFIs) are inexpensive and user-friendly diagnostic assays that can be used at the point of care (POC) [17,18]. Currently, LFIs are widely used to detect SARS-CoV-2 NP in patient samples, which potentially leads to a rapid COVID-19 diagnosis. In contrast to the spike protein, the NP is relatively conserved from variant to variant, containing only 3- and 6-amino acid changes for Delta and Omicron, respectively, in reference to the original WA1 strain.12,13 The recent cluster of Omicron subvariants, BA.2, BA.2.12.1, and BA.5 all contain the same 7-amino acid replacements in the NP, 6 of which are conserved with the BA.1 variant [19,20]. Omicron subvariant BA.4 has yet another amino acid change, resulting in 8 replacements in the NP [21]. As a consequence, detection of the NP instead of the spike protein should result in an assay that is more resilient to diagnostic escape. In fact, every rapid detection test approved under Emergency Use Authorization by the FDA since 2020 is still being recommended for SARS-CoV-2 detection [14]. The pandemic presented an unparalleled need for rapid testing. To accommodate this, companies developed rapid tests utilizing mAbs previously isolated from severe acute respiratory syndrome coronavirus (SARS-CoV) NP immunizations [22,23]. These rapid tests performed well, as the homology between SARS-CoV NP and SARS-CoV-2 NP from the WA1 strain is 90%, and the use of mAbs generated to SARS-CoV NP allowed for the rapid production of assays to detect SARS-CoV-2 NP in patient samples [24]. However, with more SARS-CoV-2 variants appearing and the relatively slow yet continuous accumulation of mutations in the NP, the need for mAbs and diagnostic tests developed specifically for SARS-CoV-2 NP may be a necessity to maintain high diagnostic efficacy.

Presented is a prototype LFI purposely designed for the detection of SARS-CoV-2 NP in clinical specimens. A library of 18 mAbs generated through immunization with the SARS-CoV-2 NP was developed. The reactivities of these mAbs were determined by enzyme-linked immunosorbent assays (ELISA). LFIs were constructed and screened using mAb pairs to develop an assay with high analytical sensitivity and minimal background signal. Following the selection of the top mAb pairs and assay optimization, the LFI prototype was tested using cultured live viruses isolated from patient nasopharyngeal swabs to determine analytical sensitivity across variants. The LFI was subsequently tested using clinical specimens preserved in viral transport media (VTM). The Abbott BinaxNOW^TM^ COVID-19 Antigen CARD was selected for direct comparison against the 1CV7/1CV14 prototype, as it was a readily available rapid test. In general, the 1CV7/1CV14 LFI achieved a higher level of detection of Omicron and Omicron subvariants, compared to the BinaxNOW^TM^ test.

## 2. Materials and Methods

### 2.1. Monoclonal Antibody Production

Female 8-week-old BALB/c mice (Charles River Laboratories, Inc., Frederick, MA, USA, RRID: IMSR_CRL:028) were immunized intraperitoneally with recombinant SARS-CoV-2 (WA1 strain) NP (The Native Antigen Company, Kidlington, UK) using Freund’s complete adjuvant (MilliporeSigma, Billerica, MA, USA). Following the initial priming injection, subsequent immunizations were performed through intraperitoneal injections of SARS-CoV-2 (WA1 strain) NP (RayBiotech, Peachtree Corners, GA, USA) mixed with Freund’s incomplete adjuvant (MilliporeSigma). Serum samples were collected through retro-orbital bleeds, and titers determined through indirect ELISA with SARS-CoV-2 (WA1 strain) NP (Thermo Fisher Scientific, Waltham, MA, USA) immobilized to the plate. Hybridoma fusions were performed using a standard protocol [25]. Monoclonal antibodies (mAbs) were purified from the cell supernatant utilizing standard protein A affinity chromatography.

### 2.2. Indirect ELISA

Microtiter 96-well flat-bottom medium binding plates (Greiner Bio-One, Kremsmünster, Austria) were coated with recombinant SARS-CoV-2 (WA1 strain) NP (Thermo Fisher Scientific) diluted in 1× DPBS (Corning, Corning, New York, NY, USA) overnight at room temperature. Plates were then washed three times with 1× PBS containing 0.05% Tween 20 (PBS-T). Plates were then blocked for 90 min at 37 °C in 1× PBS 0.5% non-fat milk and 0.1% Tween 20 (blocking buffer). Primary antibodies (mouse sera, hybridoma supernatant, or purified mAb) were diluted in blocking buffer and subsequent serially across the plate. Plates incubated in primary solution for 90 min at room temperature and were then washed three times with blocking buffer. Secondary antibody of either horseradish peroxidase-labeled polyclonal goat anti-mouse IgG antibodies (SouthernBiotech, Birmingham, AL, USA, RRID: AB_2619742) or IgG subclass-specific polyclonal goat anti-mouse antibodies (SouthernBiotech) diluted in blocking were incubated in the wells at room temperature for 60 min. Plates were washed three times with PBS-T. Tetramethylbenzidine (TMB) 2- component peroxidase substrate (SeraCare, Milford, MA, USA) was added to plates and allowed to react for 30 min at room temperature, then stopped using 1 M H_3_PO_4_. Plates were read at OD 450.

### 2.3. Lateral Flow Immunoassay (LFI) Prototype Development

The 18 purified NP-reactive mAbs were sprayed individually on CN95 nitrocellulose membranes (Sartorious, Gottingen, Germany) using a BioDotXYZ3060 dispense system (BioDot, Irvine, CA, USA) at a concentration of 1 mg/mL for the test line. Goat anti-mouse IgG (SouthernBiotech, RRID:AB_2794121) was sprayed at 1 mg/mL for the control line. Sprayed nitrocellulose was attached to the LFI backing card (DCN, Carlsbad, CA, USA) along with a CSFP203000 wicking pad (MilliporeSigma, Billerica, MA, USA). 17 of the 18 mAbs were conjugated to 40 nm gold nanoparticles (DCN, Carlsbad, CA, USA) through passive adsorption. 1CV18 was excluded from testing as a detection reagent, as the mAb failed to conjugate successfully to the gold nanoparticles. MAb pairs were evaluated in an 18 × 17 matrix to evaluate all capture and detection reagent combinations. Initial testing parameters for assessing capture/detection pairs was 100 ng/mL (positive) and 0 ng/mL (negative) of recombinant SARS-CoV-2 NP (Thermo Fisher Scientific) in 1× DPBS. An ESE Quant GOLD reader (Dialunox, Stockach, Germany) running LF Studio was used to obtain a quantitative intensity signal (mm*mV) at the test line. The signal intensity minus background values were compiled in a heat map (Supplementary Table S5). The top 10 pairs, which exhibited the highest signal over background, were chosen for further LFI development. The selected pairs underwent further testing at 1 ng/mL, 0.5 ng/mL and 0.25 ng/mL of recombinant SARS-CoV-2 (WA1 strain) NP (Thermo Fisher Scientific) in 1× DPBS. In this subsequent testing, the 1CV7 capture and 1CV14 detection combination (1CV7/1CV14) showed the highest reactivity and lowest background of the top ten pairs. As such, 1CV7/1CV14 was selected as the mAb pair for further LFI prototype development.

Initial optimization of the 1CV7/1CV14 LFI was performed with recombinant SARS-CoV-2 (WA1 strain) NP (Thermo Fisher Scientific) diluted in LFI chase buffer containing 1% F127 surfactant (QED Biosciences. San Diego, CA, USA), and 1× DPBS. These conditions were applied to conjugate pad optimization and led to the addition of the 6613H conjugate pad (Ahlstrom-Munksjö, Helsinki, Finland) to the 1CV7/1CV14 LFI. Recombinant SARS-CoV-2 (WA1 strain) NP (Thermo Fisher Scientific) diluted in the pooled nasal matrix collected from normal healthy donors, provided by InBios International Inc., Seattle, WA, USA was used to mimic clinical specimens during the optimization testing.

A panel of viral preparations were acquired from the Biodefense and Emerging Infections Research Resources Repository (BEI Resources) and analyzed on the 1CV7/1CV14 LFI. These viral preparations included rhinovirus (Catalog number NR-51447), human coronavirus (HCoV) OC43 (Catalog number NR-52725), HCoV 229E (Catalog number NR-52726), HCoV NL63 (Catalog number NR-470), Middle East respiratory syndrome Coronavirus (MERS) (Catalog number NR-50171), influenza A (Catalog number NR-19810), influenza B (Catalog number NR-44023), respiratory syncytial virus (RSV) (Catalog number NR-), and SARS-CoV (Catalog number NR-28526). In brief, 20 µL of lysate from cells infected with the respective virus were added to 130 µL of chase buffer. This solution was added to the 1CV7/1CV14 LFI and results were recorded after 20 min. All viruses in the panel were ran in duplicate. Rhinovirus, HCoV 229E, MERS, influenza A, influenza B, RSV, and SARS-CoV-2 were tested with of a 1 × 10^5^ TCID_50_/mL solution. Due to diluted stock concentrations, HCoV OC43 and HCoV NL63 were tested at 8.9 × 10^4^ TCID_50_/mL and 1.6 × 10^4^ TCID_50_/mL, respectively. SARS-CoV was tested at 1 × 10^5^ pfu/mL.

### 2.4. LFI Testing with Live SARS-CoV-2 Virus

All experiments containing live SARS-CoV-2 were conducted in a biosafety level 3 laboratory. SARS-CoV-2 variants were obtained from the BEI resources: USA-/WA1/2020, Wuhan-Hu1 equivalent; B.1.1.7 (Alpha); C.37 (Lambda); B.1.617.1 (Kappa); B.1.617.2 (Delta); and BA.1 (Omicron). Clinical specimens in the form of remnant patient nasopharyngeal swabs suspended in 1.5 mL of VTM verified via reverse transcriptase-polymerase chain reaction (RT-PCR) to be either COVID-19 positive or negative were provided by Nevada State Public Health Laboratory, Reno, NV. The Ct values of these specimens were determined through RT-PCR using the CDC influenza SARS-CoV-2 Multiplex assay [26]. Pooled normal human nasal matrix, RT-PCR confirmed negative for SARS-CoV-2, was used for contriving specimens for testing on the LFI and BinaxNOW^TM^ COVID-19 Antigen CARD (Abbott, Chicago, IL, USA). For the prototype LFI testing, a determined amount of live virus, denoted in TCID_50_/swab was diluted with pooled normal human nasal matrix to reach 20 µL of the simulated patient sample. The simulated patient sample was then mixed with 130 µL of the chase buffer, applied to the assay and allowed to resolve. Test results were recorded by two observers after 20 min, and strips were photographed inside the biological safety cabinet. The BinaxNOW^TM^ test was used as recommended (Quick Reference Instructions). Briefly, six drops of the provided chase buffer were added to the card, followed by inserting the provided nasal swab contrived with the indicated amounts (TCID_50_/swab) of the live virus in 20 µL of the nasal matrix. Results were recorded after 15 min and imaged as above. Both 1CV7/1CV14 and BinaxNOW^TM^ LFIs were evaluated with clinical specimens by taking 20 µL of VTM from about 1.5 mL of the total VTM containing nasopharyngeal swabs and adding them to the chase buffer (1CV7/1CV14 LFIs) or onto the BinaxNOW^TM^ swabs. The visible test lines (T) on the 1CV7/1CV14 LFIs and the BinaxNOW^TM^ cards were recorded as positive (+). The test lines that did not show a visible signal were recorded as negative (−). During testing of live virus not from remnant patient samples, barely visible signals were denoted as (+/−) to denote unsure. The BinaxNOW^TM^ test and the 1CV7/1CV14 LFI images were cropped to focus on the test and control lines and assembled for comparative analysis.

### 2.5. Sequence Confirmation of SARS-CoV-2 in Clinical Specimens

A fraction of the nucleic acid extracted from the VTM with nasopharyngeal swabs was subjected to SARS-CoV-2 sequencing on the Clear Labs DX platform (Clear Labs Inc., San Carlos, CA, USA) utilizing Oxford Nanopore Technology (ONT) or Illumina platform, as described in our previous work [27,28,29] The lineages of SARS-CoV-2 present in the nasopharyngeal swabs were defined by the pangolin 4.0.4; PUSHER-v1.2.133 and the sequences were submitted to the GISAID database. VTM-N1 and VTM-N6 (SARS-CoV-2 negative VTMs) were subjected to the extraction of genomic RNA for the detection of viral signatures through sequencing. The sequencing libraries were prepared using the QIAseq FX Single Cell RNA Library kit (QIAGEN, Hilden, Germany), and the SARS-CoV-2 sequences were enriched using a myBaits kit with coronavirus-specific biotinylated probes (Daicel Arbor Biosciences, Ann Arbor, MI, USA) as described previously [27]. The libraries were sequenced on NextSeq 2000, generated FASTQ files were analyzed, and the mapping reads were visualized using the SARS-CoV-2 mutations analysis tool of the QIAGEN CLC Genomics Workbench (QIAGEN, Inc., Germantown, MD, USA).

## 3. Results

### 3.1. Monoclonal Antibody Production and Reactivity

Hybridoma cell lines producing mAbs reactive with SARS-CoV-2 (WA1 strain) NP were cloned twice each by limiting dilution to ensure monoclonality and stability. Ultimately a library of 18 mAbs was created. Reactivity was verified through indirect ELISA. The IgG subclasses of all eighteen mAbs were determined by ELISA with IgG subclass specific mAbs (Supplementary Table S4). Western blot analysis was used to confirm that all eighteen mAbs were reactive to gamma-irradiated lysed Vero E6 cells infected with SARS-CoV-2 (WA1 strain).

### 3.2. 1CV7/1CV14 LFI Demonstrate no Cross-Reactivity with Tested Viruses

The 1CV7/1CV14 LFI was evaluated with a cross-reactivity panel of other respiratory viruses: rhinovirus, HCoV OC43, HCoV 229E, HCoV NL63, Middle East respiratory syndrome Coronavirus (MERS), influenza A, influenza B, respiratory syncytial virus (RSV), and SARS-CoV. No reactivity was observed during testing with the 1CV7/1CV14 prototype (Supplementary Figure S2).

### 3.3. 1CV7/1CV14 LFI and BinaxNOW^TM^ LFI Are Reactive with Multiple SARS-CoV-2 Variants

**Figure 1:**
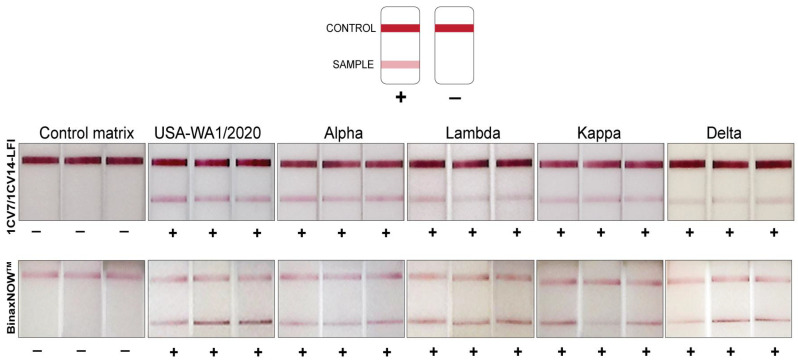
Schematic illustrating positive and negative LFI readings. Additionally, the performance of 1CV7/1CV14 LFI in comparison with BinaxNOW^TM^ for the detection of SARS-CoV-2 variants at 500 TCID_50_/test. A control matrix with known amounts of live SARS-CoV-2 virus was loaded onto the 1CV7/1CV14 LFI as well as the BinaxNOW^TM^ test in triplicates. Signals were recorded through visual inspection of the test lines.

Evaluation of the 1CV7/1CV14 LFI and BinaxNOW^TM^ LFI was performed in triplicate with live WA1, Alpha, Lambda, Kappa, and Delta SARS-CoV-2 (all classified by the CDC as variants of concern) at 500 TCID_50_ diluted into normal human nasal matrix. WA1 and the variants were consistently detected by both assays (Figure 1). Importantly, there was no background signal observed with the 1CV7/1CV14 LFI and BinaxNOW^TM^ when evaluating the normal human nasal matrix alone as a negative control.

### 3.4. 1CV7/1CV14 LFI and the BinaxNOW^TM^ LFI Have High Analytical Sensitivity for Detection of Delta and Omicron Variants

**Figure 2:**
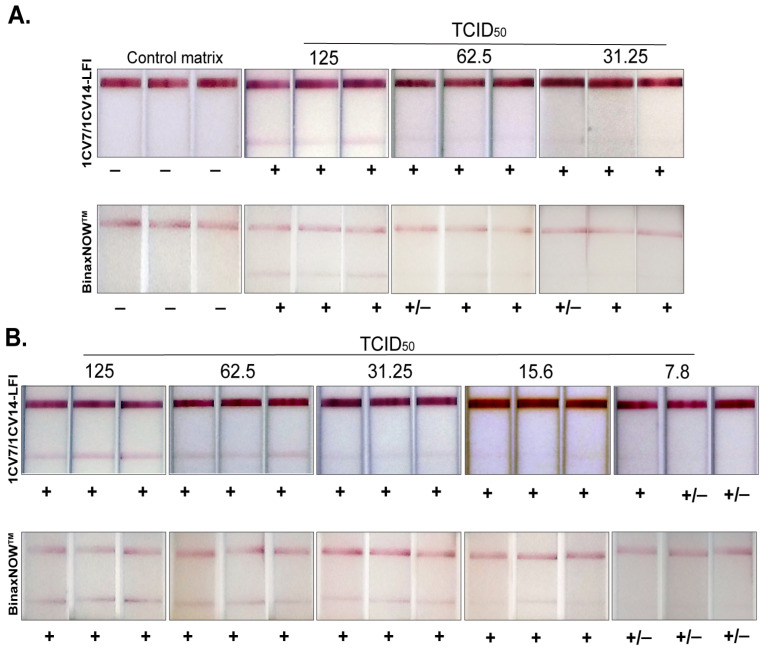
Analytical sensitivities of 1CV7/1CV14 LFI in comparison with BinaxNOW^TM^ for the detection of both Delta (**A**) and Omicron BA.1 (**B**) variants. A control matrix with known amounts of live SARS-CoV-2 virus of the respective variant was loaded onto the 1CV7/1CV14 LFI as well as the BinaxNOW^TM^ test in triplicate. Signals were recorded through visual inspection of the test lines.

Since Delta and Omicron were the predominant circulating variants at the time of this study, these two variants were used for determining LFI analytical sensitivity. The 1CV7/1CV14 LFI prototype detected the Delta variant at all three concentrations tested (125, 62.5, and 31.25 TCID_50_/swab) (Figure 2A). The BinaxNOW^TM^ tests detected the same concentrations of the Delta variant. The 1CV7/1CV14 LFI also detected the Omicron variant at 15.6 TCID_50_/swab, which was comparable to the detection limit of the BinaxNOW^TM^ test (Figure 2B). Both LFIs showed weak reactivity at 7.8 TCID_50_/swab with the Omicron variant. Normal human nasal matrix alone was evaluated on both assays in parallel as a negative control, and no background reactivity was observed (Figure 2A).

### 3.5. 1CV7/1CV14 LFI Identified More Omicron-Positive VTM Samples Than the BinaxNOW^TM^ LFI

Prior to testing Omicron RT-PCR positive samples, a panel of RT-PCR negative remnant clinical specimens, consisting of nasopharyngeal swabs in VTM collected from symptomatic individuals, were tested on the 1CV7/1CV14 and BinaxNOW^TM^ LFIs (Supplementary Figure S1). 1 RT-PCR negative sample out of the 15 was weakly positive on the 1CV7/1CV14 LFI, while none of the VTMs were positive on the BinaxNOW^TM^. Further investigation into the genomic sequences utilizing an alternative assay (described in the methods section) indicated that the false positive sample (VTM-N6) contained high levels of SARS-CoV-2 RNA, in comparison to another negative sample (VTM-N1) (Supplementary Figure S3).

**Figure 3:**
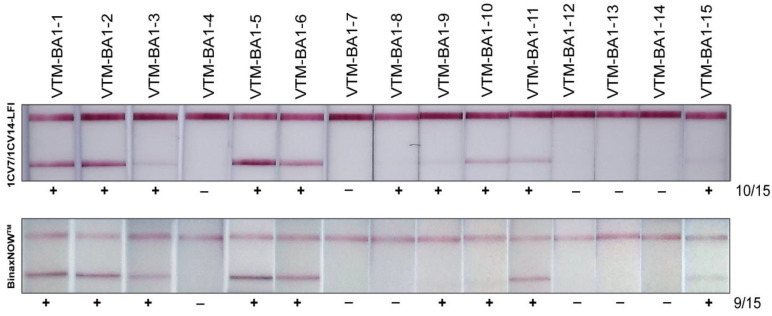
Sensitivities of the 1CV7/1CV14 LFI and BinaxNOW^TM^ in detecting Omicron (BA.1) variant in clinical samples.

**Figure 4:**
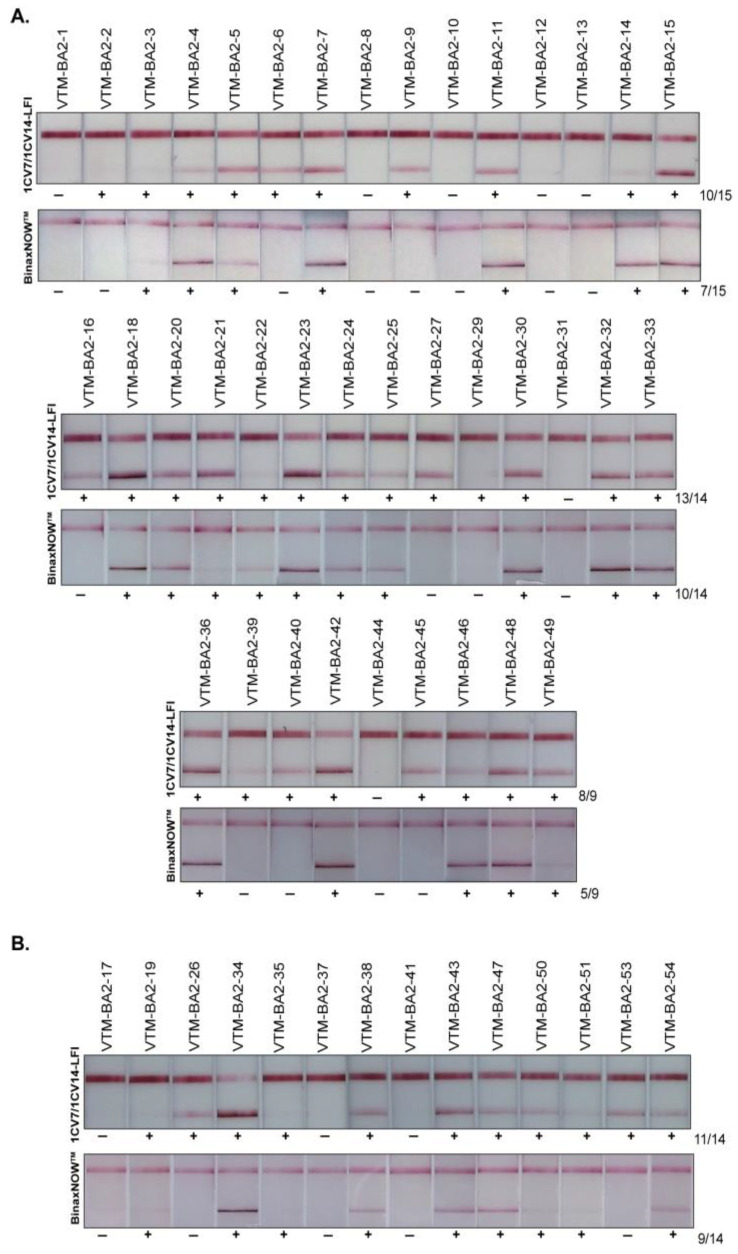
Sensitivities of 1CV7/1CV14 LFIs in comparison with BinaxNOW^TM^ for the detection of Omicron BA.2 (**A**) and BA.2.12.1 (**B**).

**Figure 5:**
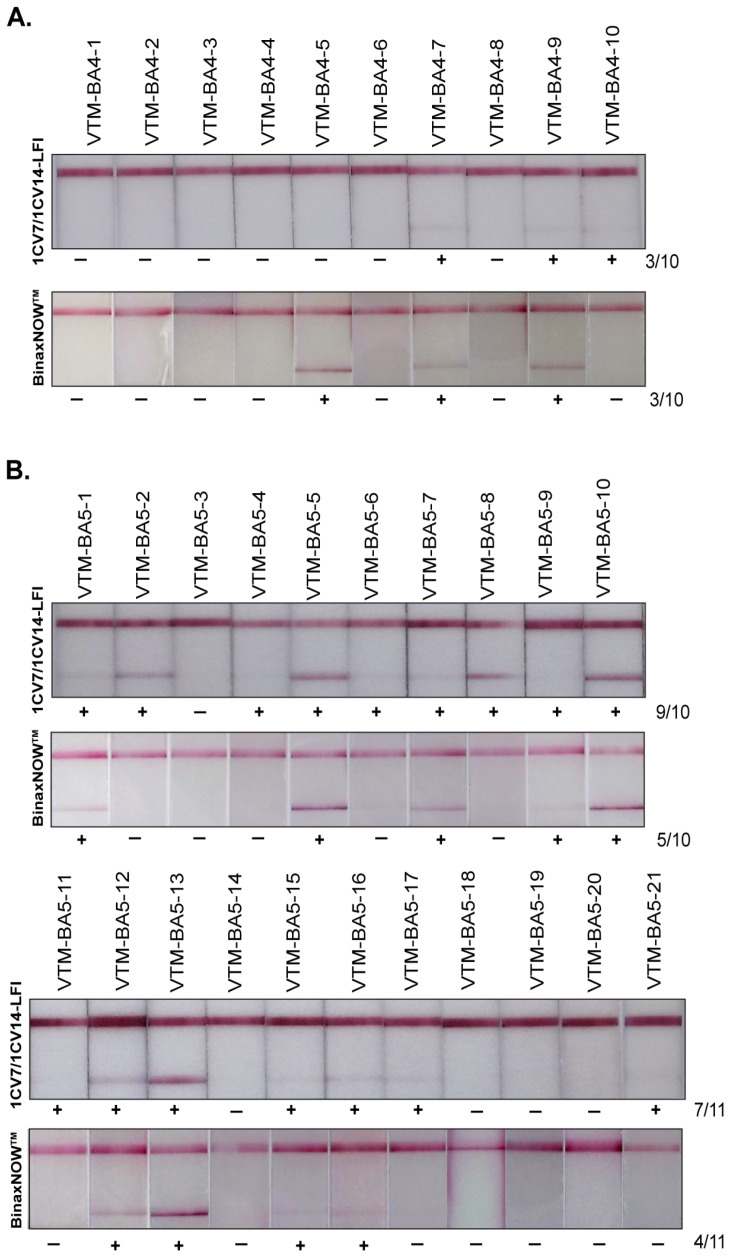
Sensitivities of 1CV7/1CV14 LFIs in comparison with BinaxNOW^TM^ for the detection of Omicron BA.4 (**A**) and BA.5 (**B**).

SARS-CoV-2 Omicron-positive remnants clinical specimens were sequenced and analyzed for lineages through NextClade [30]. Sequences of the clinical isolates were submitted to the GISAID database. A total of 99 samples confirmed to be BA.1 (Omicron), BA.2, BA.2.12.1, BA.4., and BA.5 were assayed on the 1CV7/1CV14 LFI, and results were compared with those of the BinaxNOW^TM^ test. Among 15 BA.1 specimens tested, 10 were positive on the 1CV7/1CV14 LFI and 9 were positive on the BinaxNOW^TM^ LFIs (Figure 3). The 1CV7/1CV14 performed notably better in detecting BA.2; out of the 38 BA.2 samples, 31 samples were detected on the 1CV7/1CV14 LFI compared to 22 on the BinaxNOW^TM^ test (Figure 4A). A subset of 14 samples confirmed to be BA.2.12.1 by RT-PCR (a subvariant of BA.2) resulted in 11 positive 1CV7/1CV14 tests and 9 positives with the BinaxNOW^TM^ (Figure 4B). Both the 1CV7/1CV14 LFI and BinaxNOW^TM^ test were able to detect 3 out of a total of 10 samples confirmed to be Omicron sub-variant BA.4 positive (Figure 5A). When subvariant BA.5 was tested on the two immunoassays, the 1CV7/1CV14 detected 16 samples while the BinaxNOW^TM^ detected 9, out of a total of 21 specimens (Figure 5B). We further compared the test positivity of the clinical specimens with the Ct values determined through RT-PCR using the SARS-CoV-2 and Influenza Multiplex Assay. Across all variants, the 1CV7/1CV14 LFI was able to detect SARS-CoV-2 in clinical specimens with a higher Ct value in comparison to the BinaxNOW^TM^ (Supplementary Tables S1–S3). The BinaxNOW^TM^ tests exhibited higher proportion of negative results.

## 4. Discussion

The COVID-19 pandemic has underscored the necessity for rapid, accurate, affordable diagnostics that can detect multiple SARS-CoV-2 variants. Leading commercially available rapid antigen tests in the United States are able to detect previous variants of concern [31]. However, currently circulating BA.1, BA.2, BA.2.12.1, BA.4, and BA.5 have shown reduced sensitivity in existing rapid antigen tests [32] The use of SARS-CoV-2 specific mAbs may prove beneficial for the detection of current and future variants.

In this study, mice were immunized with SARS-CoV-2 (WA1 strain) NP and the library of 18 resulting mAbs was evaluated by LFI. The best performing LFI contained mAbs 1CV7 and 1CV14. Analytical specificity testing of the 1CV7/1CV14 LFI showed no detectable cross-reactivity a small panel of respiratory viruses (Supplementary Figure S2). Notably, the 1CV7/1CV14 LFI did not react with irradiated lysate from SARS-CoV infected cells, indicating our mAbs are recognizing a SARS-CoV-2 NP epitope that is not present on the SARS-CoV NP.

When tested with live virus combined with normal human nasal matrix, at a singular concentration of 500 TCID_50_/test, the 1CV7/1CV14 prototype LFI was able to detect WA1, Alpha, Lambda, Kappa, Delta, and Omicron variants of SARS-CoV-2. Due to Omicron being the most prevalent variant circulating at time of this study, reactivity to this variant was the main focus. The 1CV7/1CV14 LFI achieved an analytical LOD of 15.6 TCID_50_ when testing cultured virus. This was evaluated against the BinaxNOW^TM^ test, which showed a similar LOD (Figure 2B). This is consistent with the two LFIs’ similar test positivity rates with remnant RT-PCR-positive Omicron BA.1 clinical specimens (Figure 2).

When testing remnant, RT-PCR confirmed, Omicron BA.2 or BA.2.12.1 positive patient specimens the 1CV7/1CV14 LFI was able to detect more (42 of 52) of positive specimens than the BinaxNOW^TM^ test (31 of 52) (Table 1). Also consistent with this, the remnant RT-PCR-positive BA.2 or BA.2.12.1 specimens that were detected as positive by the 1CV7/1CV14 LFI had higher RT-PCR Ct values than those that the BinaxNOW^TM^ test was able to detect. The same pattern of greater test positivity rate and detection of specimens with higher Ct values by the 1CV7/1CV14 LFI was also seen with the RT-PCR-positive BA.5 remnant specimens. Additionally, the test lines on the 1CV7/1CV14 LFI appeared more intense, potentially providing easier identification of positive tests, and hence positive specimens. This could be due to mAbs 1CV7 and 1CV14 recognizing more conserved epitopes, or the mAbs simply bind with a higher affinity.

Regarding the detection of BA.4 in clinical samples, both the 1CV7/1CV14 LFI and the BinaxNOW^TM^ test detected 3 of 10 samples (Figure 5A, Table 1). BA.4 has a unique P151S replacement, in comparison to all other variants [21]. It is possible that the amino acid change from proline to serine results in a slight conformational change in the NP, decreasing the binding ability of the 1CV7, 1CV14, and the BinaxNOW^TM^ antibodies.

Overall, the data presented here demonstrate that the 1CV7/1CV14 LFI is highly inclusive of multiple SARS-CoV-2 strains. Furthermore, the 1CV7/1CV14 LFI appears to be more sensitive in detecting of BA.1, BA.2, BA.2.12.1, and BA.5 in remnant patient specimens when directly compared to the BinaxNOW^TM^ test. The 1CV7/1CV14 LFI detected 71 of 98 samples while the BinaxNOW^TM^ test detected only 52 of the 98 samples (Table 1). The data from this study suggest that the use of SARS-CoV-2 specific mAbs can result in a more efficacious immunoassay in detecting Omicron and Omicron subvariants. This study was limited in the use of patient nasopharyngeal swabs suspended in VTM. While the use of remnant clinical samples is a start, future studies should be oriented toward the utilization of fresh patient nasopharyngeal swabs. The analytical sensitivity of the 1CV7/1CV14 LFI could potentially be further enhanced by (i) adding additional capture or detection mAbs from the existing mAb library (or from other mAb libraries), (ii) improving the composition of the chase buffer, and/or (iii) the altering the components of the LFI. Furthermore, this study draws attention to the potential importance of developing rapid diagnostic assays with SARS-CoV-2 derived mAbs to hopefully achieve high inclusivity (and hence long-term diagnostic efficacy) across newly evolved SARS-CoV-2 variants of concern.

## Figures and Tables

**Table 1:** Summary of 1CV7/1CV14 LFI and BinaxNOW^TM^ Ag Card SARS-CoV-2 variant testing as seen in Figure 3, Figure 4 and Figure 5.

Variant	Total Specimens	1CV7/1CV14 LFI Positives	BinaxNOW^TM^ Ag Card Positives
BA.1	15	10	9
BA.2	38	31	22
BA.2.12.1	14	11	9
BA.4	10	3	3
BA.5	21	16	9
Total	98	71	52

## Data Availability

The data presented in this manuscript are available from the corresponding author upon reasonable request.

## Supplementary Materials

The following supporting information can be downloaded at: https://www.mdpi.com/article/10.3390/v14122609/s1, Figure S1: testing of rt-PCR negative clinical specimens preserved in VTM with the 1CV7/1CV14 LFI and BinaxNOW^TM^ (20 µL sample/test); Figure S2: cross-reactivity testing of 1CV7/1CV14 LFI. Rhinovirus, HCoV 229E, MERS, influenza A, influenza B, RSV, and SARS-CoV-2 concentrations are 1 × 10^5^ TCID_50_/mL. Due to diluted stock concentrations, HCoV OC43 and HCoV NL63 are at 8.9 × 10^4^ TCID_50_/mL and 1.6 × 10^4^ TCID_50_/mL, respectively. SARS-CoV was tested at 1 × 10^5^ pfu/mL; Figure S3: sequencing of the genomic RNA from the nasopharyngeal swabs of these two specimens showed high levels of the SARS-CoV-2 genome in VTM-N6 as compared to minimal reads in another SARS-CoV-2 negative specimen (VTM-N1); Table S1: all BA.1 clinical specimens with corresponding Ct values; Table S2: all BA.2 and sub-variant BA.2 clinical specimens with corresponding Ct values; Table S3: all BA.4 and BA.5 clinical specimens with corresponding Ct values; Table S4: all mAbs generated for mAb library. Subclass is indicated as well as reactivity toward both recombinant NP and viral lysate: “+” indicating reactive and “−” indicating non-reactive; Table S5: all by all testing of capture/detection pairs. Darker green indicates a higher difference between signal and background.
