# Supplementary material for: Comparison of a Prototype SARS-CoV-2 Lateral Flow IMMUNOASSAY with the BinaxNOWTM COVID-19 Antigen CARD
Source: Viruses. 2022 Nov 23;14(12):2609. doi: 10.3390/v14122609 (PMC9786212; doi:10.3390/v14122609)

Supplementary Figures

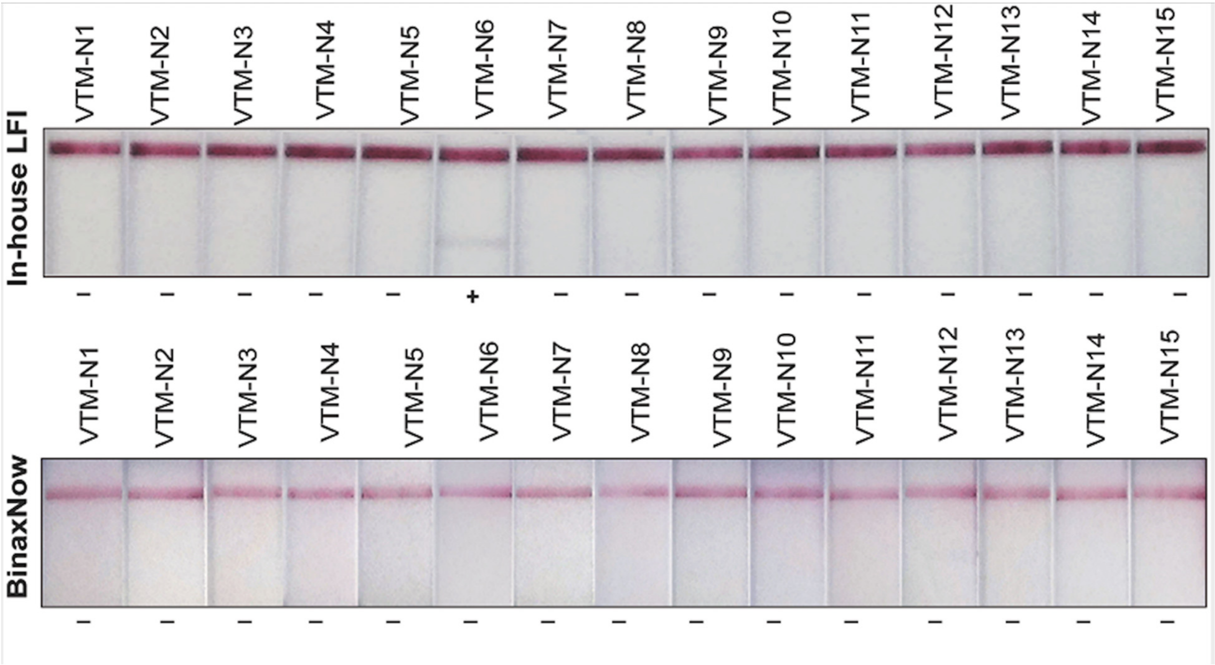

Figure S1. Testing of rt-PCR negative clinical specimens preserved in VTM with the 1CV7/1CV14 LFI and BinaxNOW™ (20 μL sample/test)

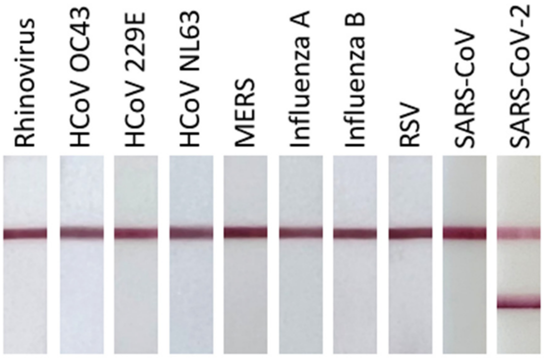

Figure S2. Cross-reactivity testing of 1CV7/1CV14 LFI. Rhinovirus, HCoV 229E, MERS, influenza A, influenza B, RSV, and SARS-CoV-2 concentrations are 1x10^5 TCID$_{50}$/mL. Due to diluted stock concentrations, HCoV OC43 and HCoV NL63 are at 8.9x10^4 TCID$_{50}$/mL and 1.6x10^4 TCID$_{50}$/mL, respectively. SARS-CoV was tested at 1x10^5 pfu/mL.

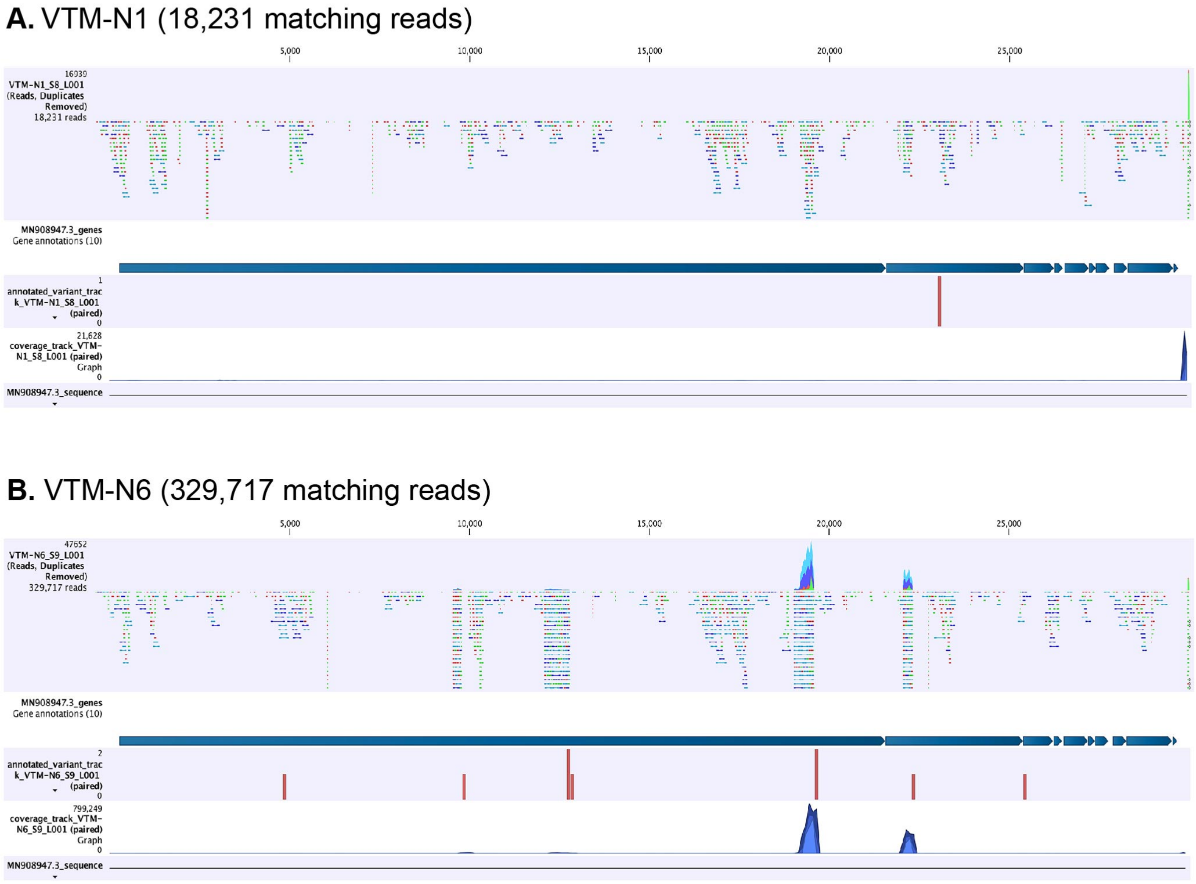

Figure S3. Sequencing of the genomic RNA from the nasopharyngeal swabs of these two specimens showed high levels of the SARS-CoV-2 genome in VTM-N6 as compared to minimal reads in another SARS-CoV-2 negative specimen (VTM-N1).

Table S1. All BA.1 clinical specimens with corresponding Ct values.

UNR sample IDs	pango_lineage	Ct values
VTM-BA1-1	BA.1.1	25.09
VTM-BA1-2	BA.1.1	24.32
VTM-BA1-3	BA.1.1	26.25
VTM-BA1-4	BA.1.1	30.06
VTM-BA1-5	BA.1.1	20.43
VTM-BA1-6	BA.1.1	25.66
VTM-BA1-7	BA.1.1.1	35.54
VTM-BA1-8	BA.1	32.36
VTM-BA1-9	BA.1.1	26.86
VTM-BA1-10	BA.1.15	29.56
VTM-BA1-11	BA.1.1	26.57
VTM-BA1-12	BA.1	27.79
VTM-BA1-13	BA.1.1	27.84
VTM-BA1-14	BA.1	28.67
VTM-BA1-15	BA.1	28.82

Table S2. All BA.2 and sub-variant BA.2 clinical specimens with corresponding Ct values.

UNR Sample ID	pango_lineage	Ct values
VTM-BA2-1	BA.2	23.998
VTM-BA2-2	BA.2	26.182
VTM-BA2-3	BA.2	22.799
VTM-BA2-4	BA.2	15.164
VTM-BA2-5	BA.2	21.678
VTM-BA2-6	BA.2	31.838
VTM-BA2-7	BA.2.3	24.763
VTM-BA2-8	BA.2	30.051
VTM-BA2-9	BA.2	26.558
VTM-BA2-10	BA.2.3	24.626
VTM-BA2-11	BA.2	15.106
VTM-BA2-12	BA.2	19.881
VTM-BA2-13	BA.2	25.742
VTM-BA2-14	BA.2	14.989
VTM-BA2-15	BA.2	20.665
VTM-BA2-16	BA.2.9	23.163
VTM-BA2-18	BA.2.9	15.458
VTM-BA2-20	BA.2	16.75
VTM-BA2-21	BA.2.3	21.354
VTM-BA2-22	BA.2.3	17.214
VTM-BA2-23	BA.2	14.768
VTM-BA2-24	BA.2.9	16.405
VTM-BA2-25	BA.2	16.905
VTM-BA2-27	BA.2	22.68
VTM-BA2-29	BA.2	21.115
VTM-BA2-30	BA.2.3	14.832
VTM-BA2-31	BA.2	28.222
VTM-BA2-32	BA.2	15.229
VTM-BA2-33	BA.2	15.371
VTM-BA2-36	BA.2	15.158
VTM-BA2-39	BA.2.3	26.823
VTM-BA2-40	BA.2	20.231
VTM-BA2-42	BA.2	14.301
VTM-BA2-44	BA.2	22.529
VTM-BA2-45	BA.2	26.262

VTM-BA2-46	BA.2.10	17.963
VTM-BA2-48	BA.2	17.177
VTM-BA2-49	BA.3	21.122
VTM-BA2-17	BA.2.12.1	25.148
VTM-BA2-19	BA.2.12.1	19.917
VTM-BA2-26	BA.2.12.1	20.486
VTM-BA2-34	BA.2.12.1	14.365
VTM-BA2-35	BA.2.12.1	17.353
VTM-BA2-37	BA.2.12.1	26.699
VTM-BA2-38	BA.2.12.1	18.873
VTM-BA2-41	BA.2.12.1	27.925
VTM-BA2-43	BA.2.12.1	18.31
VTM-BA2-47	BA.2.12.1	16.462
VTM-BA2-50	BA.2.12.1	16.888
VTM-BA2-51	BA.2.12.1	19.563
VTM-BA2-53	BA.2.12.1	17.416
VTM-BA2-54	BA.2.12.1	15.185

Table S3. All BA.4 and BA.5 clinical specimens with corresponding Ct values.

UNR Sample ID	pango_lineage	Ct values
VTM-BA4-1	BA.4	28.893
VTM-BA4-2	BA.4	25.777
VTM-BA4-3	BA.4	24.901
VTM-BA4-4	BA.4	26.409
VTM-BA4-5	BA.4	18.941
VTM-BA4-6	BA.4	27.915
VTM-BA4-7	BA.4	16.405
VTM-BA4-8	BA.4	21.541
VTM-BA4-9	BA.4	16.989
VTM-BA4-10	BA.4	27.405
VTM-BA5-1	BA.5	18.466
VTM-BA5-2	BA.5	28.567
VTM-BA5-3	BA.5	31.162
VTM-BA5-4	BA.5	26.698
VTM-BA5-5	BA.5	15.617
VTM-BA5-6	BA.5	17.32
VTM-BA5-7	BA.5	18.398
VTM-BA5-8	BA.5	19.34
VTM-BA5-9	BA.5	17.601
VTM-BA5-10	BA.5	14.826
VTM-BA5-11	BA.5	17.25
VTM-BA5-12	BA.5	18.786
VTM-BA5-13	BA.5	15.14
VTM-BA5-14	BA.5	26.569
VTM-BA5-15	BA.5	19.405
VTM-BA5-16	BA.5	19.493
VTM-BA5-17	BA.5	22.089
VTM-BA5-18	BA.5	21.489
VTM-BA5-19	BA.5	27.24
VTM-BA5-20	BA.5	27.457
VTM-BA5-21	BA.5	28.65

Table S4. All mAbs generated for mAb library. Subclass is indicated as well as reactivity toward both recombinant NP and viral lysate: "+" indicating reactive and "-" indicating non-reactive.

Clone	Subclass	Reactivity to recombinant NP (+/-)	Reactivity to inactivated virus (+/-)
**1CV1**	IgG2a	+	+
**1CV2**	IgG1	+	+
**1CV3**	IgG1	+	+
**1CV4**	IgG1	+	+
**1CV5**	IgG1	+	+
**1CV6**	IgG2a	+	+
**1CV7**	IgG1	+	+
**1CV8**	IgG1	+	+
**1CV9**	IgG2b	+	+
**1CV10**	IgG1	+	+
**1CV11**	IgG1	+	+
**1CV12**	IgG1	+	+
**1CV13**	IgG1	+	+
**1CV14**	IgG2b	+	+
**1CV15**	IgG2b	+	+
**1CV16**	IgG1	+	+
**1CV17**	IgG1	+	+
**1CV18**	IgG1	+	-

Table S5. All by all testing of capture/detection pairs. Darker green indicates a higher difference between signal and background.

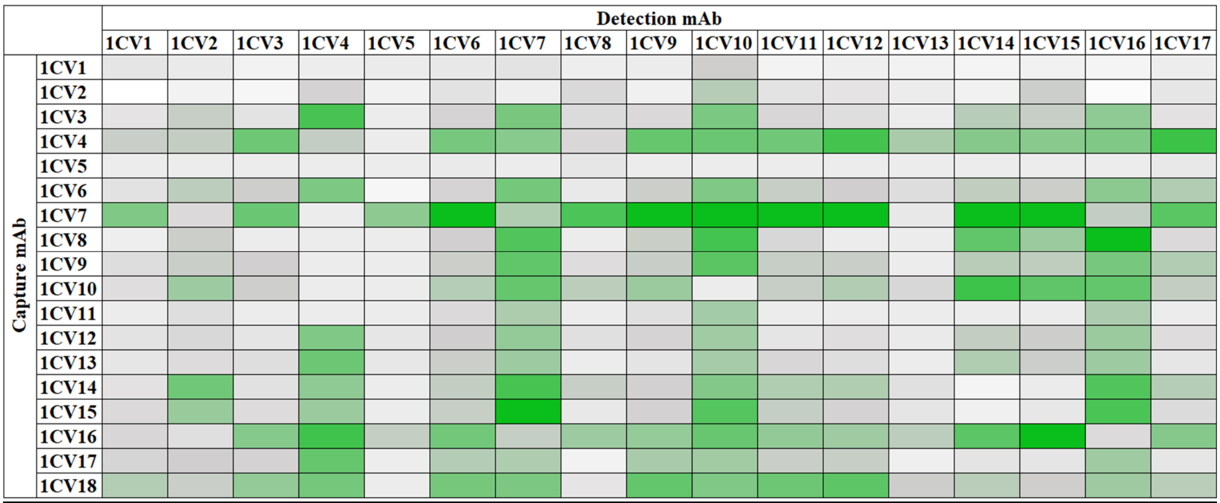

Supplement: Supplementary file 1 [file viruses-14-02609-s001.zip › viruses-2036959-SI.pdf]
